# Steps toward developing an algorithm to facilitate recognition of translational science

**DOI:** 10.1017/cts.2026.10753

**Published:** 2026-05-15

**Authors:** Paul J. Martin, Brian E. Saelens, Theodore Johnson, Mark Harniss, Tumaini R. Coker, Erin Abu-Rish Blakeney, Nina Isoherranen, Tong Sun, Nisha Bansal, Stephanie J. Lee, John Amory, Naomi Kaku, Melissa Vaught

**Affiliations:** 1 Institute of Translational Health Sciences, University of Washington, Seattle, WA, USA; 2 Fred Hutchinson Cancer Center, Seattle, WA, USA; 3 Medicine, University of Washington, Seattle, WA, USA; 4 Seattle Children’s Hospital, Seattle, WA, USA; 5 Pediatrics, University of Washington, Seattle, WA, USA; 6 Institute on Human Development and Disability, University of Washington, Seattle, WA, USA; 7 Rehabilitation Medicine, University of Washington, Seattle, WA, USA; 8 Biobehavioral Nursing and Health Informatics, University of Washington, Seattle, WA, USA; 9 Pharmaceutics, University of Washington, Seattle, WA, USA; 10 Plant Sciences and Plant Pathology, Montana State University, Bozeman, MT, USA

**Keywords:** Translational science, translational research, clinical and translational science award, hub operations, research assessment

## Abstract

**Introduction:**

In preparing for our Clinical and Translational Science Award (CTSA) UM1 application, we recognized the need to develop a shared understanding of the distinctions between translational science (TS) and translational research (TR). We describe our efforts to develop and evaluate the reliability of a concise instrument that investigators and reviewers could use to distinguish between TS and TR.

**Methods:**

Groups of faculty and staff individually reviewed published translational studies to determine whether the project involved TS and, separately, TR. One group (*n* = 10) first reviewed 14 publications with limited guidance; the same group and a second group (*n* = 9) then reviewed another set of 14 publications guided by a detailed algorithm. We used kappa statistics to measure agreement in the determinations of TS and TR for each publication.

**Results:**

The overall kappa coefficients in the three sets of TS determinations (two by the first group and one by the second group) were 0.61, 0.33, and 0.18, respectively. The overall kappa coefficients in the three sets of TR determinations were 0.26, 0.11, and 0.40, respectively. The median kappa coefficients for all 42 determinations were 0.39 for TS and 0.22 for TR, both indicating only fair agreement. We found no evidence that the algorithm helped to improve agreement rates.

**Conclusion:**

Our results show gaps in understanding the distinction between TS and TR among CTSA hub faculty and staff. We discuss some reasons for this gap and propose ways that could improve the recognition of TS and TR.

## Introduction

Translational science (TS) is an emerging discipline that seeks to deliver evidence-based approaches that improve scientific and operational quality and efficiency in clinical research and translational research (TR). In its latest iteration, the National Center for Advancing Translational Sciences (NCATS) signaled a stronger requirement to advance TS in the Clinical and Translational Science Award (CTSA) programs [1,2]. In the current UM1, pilot awards must support TS, and the application must include a new research program (Element E) that supports discrete projects addressing significant TS roadblocks.

According to the current UM1 notice of funding opportunity (NOFO) for CTSAs (PAR-24-272 [2]), translation is “the process of turning observations in the laboratory, clinic and community into interventions that improve the health of individuals and communities – from diagnostics, preventions, and treatments to medical procedures and behavioral changes.” The UM1 NOFO defines TR as “the endeavor to traverse a particular step of the translational process for a particular target or disease.” It defines TS as “the field that addresses longstanding scientific and operational challenges along the translational science spectrum through innovations that transform the way research is conducted, making it faster, more efficient, and more impactful.”

In 2022, NCATS staff published an initial set of guiding principles for TS generated via case studies of successful TS initiatives [3,4]. Briefly summarized, effective TS projects a) prioritize initiatives that address unmet needs, b) produce generalizable solutions for common and persistent challenges, c) emphasize creativity and innovation, d) leverage cross-disciplinary team science, e) enhance the efficiency and speed of TR, and f) use bold and rigorous research approaches [3]. NCATS later added g) prioritize diversity, equity, inclusion, and accessibility [5], although this is not currently included on the active page [4]. NCATS acknowledged that these principles apply to translation broadly in both TR and TS and were not entirely focused on distinguishing between the two.

Within our own CTSA program (the Institute of Translational Health Sciences, or ITHS), the lack of clarity became evident in January 2024, when we issued a request for ideas that could be developed into Element E projects for our UM1 application. Many of these proposals used the above principles to suggest that the work would involve TS. Yet after careful consideration, the reviewers concluded that most of the proposals involved TR with little if any TS. Therefore, we sought to develop a shared understanding of the distinctions between TS and TR to identify and describe our TS contributions, to develop research projects that will advance TS, and to deliver and communicate the impact of these contributions to our hub partners, collaborating institutions, and NCATS. We recognized a need for education and training for both internal and external audiences to encourage development of future translational scientists who will work to improve how health research is designed, done, and disseminated. Here, we describe our efforts to develop and evaluate the reliability of a concise instrument that investigators and reviewers could use to distinguish between TS and TR. We propose an approach to distinguish between TS and TR for further testing and evaluation.

## Methods

### Selection of publications and participants

In this study, we used publications rather than project applications because publications are in the public domain, are not subject to concerns about intellectual property, and have gone through peer review. We compiled a source set of 282 publications that acknowledged our ITHS UL1 grant as a source of support and were reported in our ITHS research performance progress reports between 2018 and 2023, before NCATS mandated a focus on TS. Publications from our KL2 and TL1 programs were not considered unless they also acknowledged the UL1 award. To enrich for TS studies, we considered reports by authors who received effort from the UL1 award and excluded publications that cited the UL1 solely due to ITHS pilot funding or resource sharing.

Publication titles were screened informally by PJM and MV to make provisional assessments of whether the studies could be considered translational and could have involved TS or not, based on NCATS principles and definitions [1,5], yielding 31 publications. Disagreements were discussed with the goal of identifying challenging areas for distinguishing TS. PJM and MV provisionally categorized publications as reporting TS alone, TR alone, or both TS and TR and then categorized by subject matter as biostatistics, representative cohort selection, electronic health records (EHR), ethics, or other. From this process, we selected 20 publications to serve as review material for the project, primarily removing some EHR and ethics publications, which were overrepresented compared to other categories.

One included publication arose from a pilot award project [6], funded by a solicitation that specifically supported development of tools and resources that could be generalized to other TR projects and fields. The remaining publications resulted from research, consultation, or service by an ITHS program (e.g., Biomedical Informatics, Community Engagement) [7–25]. Since none of the 282 publications in the source set involved early stage translational laboratory work, PJM screened recent issues of the journal *Science* and selected 8 publications that clearly had translational relevance [26–33]. We used stratified randomization by primary subject matter category to divide the 28 publications into Set 1 and Set 2, each containing 14 publications. During a regularly scheduled online meeting, the ITHS Steering Committee members were invited to participate in the study, and 19 individuals volunteered to assess the publications.

### Study design

The University of Washington Human Subjects Division determined that this research qualified for exempt status (Categories 2 and 3). We randomly assigned the 19 study participants to 2 groups designated Group A and Group B. In Round 1 of the project, we asked the 10 members of Group A to determine individually whether the publications in Set 1 involved TS (Yes/No) and whether they involved TR (Yes/No), guided by a letter that simply quoted the current NCATS definition of TS, updated in September 2024 [2] (Supplemental File S1), and emphasized that the categories are not mutually exclusive. In addition, the participants used a 5-point Likert scale to rate their level of confidence that their determination of TS and TR in each publication would agree with the determinations of other reviewers. Scores ranged from 1 to 5 respectively expressing doubt, low confidence, moderate confidence, good confidence, or high confidence.

After the results were returned, PJM and MV hosted two 1-hour discussion groups for Group A participants to review the results and to assess a draft algorithm designed to provide more extensive guidance regarding the distinction between TS and TR. Four to five participants joined each meeting, with no overlap; one individual was unable to participate in either meeting. Discussion was guided by open-ended questions of TS and TR determinations, with an emphasis on divergent responses rather than those achieving consensus. We also provided a draft algorithm and asked for input on perceptions of its utility and potential improvements. The algorithm was initially developed by PJM through a careful review of the contemporaneous guiding principles published by NCATS [5], modified to remove principles shared between TS and TR and to emphasize the features that distinguish TS from TR. Results of this discussion produced an algorithm for subsequent testing (Supplemental File S2).

In Round 2 of the project, we sent a cover letter (Supplemental File S1) to the members of Groups A and B asking them to determine individually whether the publications in Set 2 involved TS and whether they involved TR, guided by the algorithm. The participants also used the 5-point Likert scale to rate their level of confidence in the determinations of TS and TR in each publication.

Finding lower agreement in TS and TR determinations in Round 2, when participants were provided an algorithm, we elected to conduct one-on-one interviews with participants who were more frequently among the minority, particularly for TS determinations. PJM prepared a summary for each publication to illustrate how application of the algorithm would yield the majority determination. MV and PJM developed an interview guide with questions pertinent to motivations for participation, prior experience, and algorithm review (Supplemental File S3). PJM conducted interviews (approximately 30 minutes each) with six participants.

### Statistics

For each publication, the participants scored the presence of TS as “Y” and its absence as “N.” We calculated percent agreement between reviewers and the free-marginal kappa statistic [34] in their determinations of TS for each publication. This analysis considers all pairwise combinations of reviewers (e.g., 10 reviewers yield 45 pairwise combinations per publication). We used the same methods to measure agreement in the determinations of TR for each publication. A kappa coefficient less than 0 indicates less agreement than would be expected by chance given a large sample, while a score of 0 indicates the level of agreement that would be expected purely by chance. As a rule of thumb, kappa coefficients in the following ranges can be interpreted as agreement better than expected by chance: from 0.01 to 0.20 (slight), 0.21 to 0.40 (fair), 0.41 to 0.60 (moderate), 0.61 to 0.80 (substantial), and 0.81 to 1.0 (almost perfect) [35].

Confidence scores for the TS and TR determinations are reported as means. We hypothesized that for Group A, agreement in the TS and TR determinations would be higher in Round 2 than in Round 1 and that for Group B, agreement would be higher in Round 2 than it had been for Group A in Round 1. We used the rank-sum test to assess differences in kappa coefficients and confidence ratings between group and set combinations. For all comparisons, an unadjusted two-sided *p*-value of <0.05 was considered statistically significant.

## Results

According to definitions of the *Journal of Clinical and Translational Science*, 21 of the 28 publications selected for determination of TS and TR involved original research (Supplemental File S4). Other study types included a commentary, an original research plan, a perspective, a review, and three position papers. The primary subject matter categories of the publications were biostatistics (*N* = 3), representative cohort selection (*N* = 3), use of electronic health records (*N* = 6), ethics (*N* = 6), laboratory studies (*N* = 8), and other (*N* = 2). Table 1 summarizes the characteristics of the study volunteers in Groups A and B. The participants included faculty (*N* = 14) and staff (*N* = 5) with between 5 and 45 years of professional experience encompassing a broad range of translational expertise and clinical research disciplines, although only one of the participants had extensive laboratory experience. Five participants had previously served as reviewers for the ITHS Pilots Program. One participant in Group A completed only the first half of the study.

**Table 1.** Characteristics of study participants Table 1 long description.

Category	All participants	Group A	Group B
***Role, N***			
Staff	5	2	3
Faculty	14	8	6
*Translational stage, N* ^*^			
Preclinical	4	4	0
Clinical Research	13	8	5
Clinical Implementation	10	5	5
Public Health	5	3	2
Other (Medical Education)	1	0	1
*Research discipline, N* ^*^			
Method or process development	4	2	2
Translation from basic to clinical research	1	1	0
Biomedical or health informatics	1	0	1
Health outcomes, services, effectiveness	5	2	3
Clinical epidemiology	1	1	0
Clinical trials	11	7	4
Digital health, mobile health, social media	1	0	1
Pediatrics	5	3	2
Behavioral or social science	5	3	2
Other (Research IT)	1	1	0
***Years of Experience, Mean*** ± ***SD (range)***	21 ± 11 (5 to 45)	22 ± 14 (6 to 45)	19 ± 7 (5 to 27)

* Participants were allowed to select all categories that applied.

### Determinations of TS

**Figure 1.**
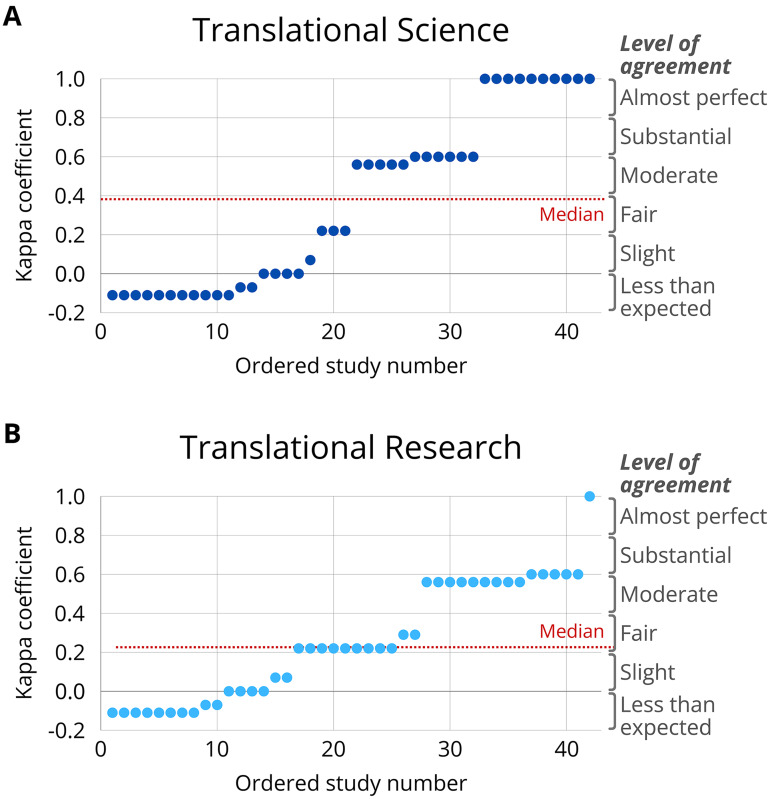
Figure 1 long description. Distribution of kappa coefficients for TS and TR assessments. The 42 assessments of publications in the study (28 for Group A, 14 for Group B) are plotted in order according to their kappa coefficients for (A) TS and (B) TR. The red dashed lines indicate the median kappa coefficient across all assessments in the category. Right-side labels denote conventional terminology for the interpretation of kappa coefficients.

Determinations of TS and TR in each publication by each participant are summarized in Supplemental File S4. Contrary to expectation, agreement in the determinations of TS by Group A was lower for Set 2 (median kappa = 0.22) than for Set 1 (median kappa = 0.60; *p* = 0.04) (Table 2). Agreement in the determinations of TS for Set 2 by Group B (median kappa = −0.06) did not differ statistically from those by Group A (*p* = 0.28). Across all participants in both groups, kappa coefficients reached or surpassed 0.60, indicating nearly substantial or substantial agreement in the determination of TS in 16 (38%) of the 42 assessments of the publications in Sets 1 and 2 (Figure 1(A)). The median kappa score across all 42 assessments of TS was 0.39, indicating fair agreement. The overall kappa coefficients of TS determinations for Group A Sets 1 and 2 and Group B Set 2 were 0.61, 0.33, and 0.18, respectively (Table 2).

**Table 2.** Agreement and confidence scores for determination of TS and TR Table 2 long description.

Outcome	Group A Set 1	Group A Set 2	*p*-Value^*^	Group B Set 2	*p*-Value^†^
Percent TS agreement, median	80	55.5	0.04	47	0.28
TS kappa, median	0.60	0.22		−0.06	
Percent TS agreement, overall	80	67		59	
TS kappa, overall	0.61	0.33		0.18	
TS confidence score, mean	3.72	4.25	<0.001	3.86	0.005
Percent TR agreement, median	62.5	50	0.11	78	0.006
TR kappa, median	0.26	0.0		0.56	
Percent TR agreement, overall	63	56		70	
TR kappa, overall	0.26	0.11		0.40	
TR confidence score, mean	4.03	4.38	0.01	3.86	<0.001

* Group A Set 1 vs. Group A Set 2 (for median kappa and confidence score).

† Group A Set 2 vs. Group B Set 2 (for median kappa and confidence score).

Despite lower agreement, TS confidence scores in Group A were higher for Set 2 (mean, 4.25) than for Set 1 (mean, 3.72; *p* < 0.001, rank-sum test). TS confidence scores for Set 2 were lower in Group B (mean, 3.86) than in Group A (mean, 4.25; *p* = 0.005, rank-sum test).

### Determinations of TR

Agreement in the determinations of TR by Group A did not differ statistically between Set 1 (median kappa = 0.26) and Set 2 (median kappa = 0.0; *p* = 0.11). Agreement in the determinations of TR for Set 2 by Group B (median kappa = 0.56) was higher than the agreement rate in Group A (*p* = 0.006). Across all participants in both groups, kappa coefficients in the determination of TR reached or surpassed 0.60 in 6 (14%) of the 42 assessments of publications in Sets 1 and 2, with lower levels of agreement in the other 36 assessments (Figure 1(B)). The median kappa score across all 42 assessments of TR was 0.22, indicating fair agreement. The overall kappa coefficients of TR determinations for Group A Sets 1 and 2 and Group B Set 2 were 0.26, 0.11, and 0.40, respectively (Table 2).

TR confidence scores in Group A were higher for Set 2 (mean, 4.38) than for Set 1 (mean, 4.03; *p* = 0.01). Confidence scores for Set 2 were lower in Group B (mean, 3.86) than in Group A (*p* ≤ 0.001).

To assess possible differences in agreement rates between faculty and staff, we evaluated results for faculty members as a separate group. This exploratory analysis did not show major differences for either TS or TR assessments when compared to the overall group (Supplemental File S5).

### Agreement according to publication subject matter

Across both Sets 1 and 2 and Groups A and B, agreement in the determinations of TS was lower for laboratory-based publications than for those in other subject matter categories (Table 3). Agreement rates were less than 60% in 11 of the 12 assessments of laboratory studies in Sets 1 and 2, and the median kappa score for the 12 assessments was −0.11. Median agreement rates in the determinations of TR across the subject matter categories were spread between 47% and 78%, and the median kappa coefficients ranged between −0.07 and 0.56, indicating no better than moderate agreement. Across both Sets 1 and 2 and Groups A and B, the median kappa coefficients in the determination of TS and TR did not differ statistically between publications presenting original research versus those not representing original research (0.56 vs. 0.22 for TS, *p* = 0.77; 0.22 vs. 0.56 for TR, *p* = 0.09).

**Table 3.** Median agreement and kappa coefficients according to publication subject matter Table 3 long description.

		Translational science		Translational research	
Publication subject matter	N^*^	Agreement (range)	Kappa (range)	Agreement (range)	Kappa (range)
Biostatistics	3	100 (100)	1.0 (1.0 to 1.0)	61 (44–78)	0.22 (−0.11 to 0.56)
EHR	6	80 (50–100)	0.60 (0 to 1.0)	53 (44–78)	0.07 (−0.11 to 0.56)
RCS	3	78 (44–100)	0.56 (−0.11 to 1.0)	65.5 (44–80)	0.32 (−0.11 to 0.60)
Ethics	6	61 (44–100)	0.22 (−0.11 to 1.0)	78 (44–80)	0.56 (−0.11 to 0.60)
Laboratory	8	44 (44–80)	−0.11 (−0.11 to 0.6)	62.5 (47–100)	0.26 (−0.07 to 1.0)
Other	2	78 (50–100)	0.56 (0 to 1.0)	47 (44–61)	−0.07 (−0.11 to 0.22)

RCS = representative cohort selection, EHR = electronic health records.

* Set 1 publications (Group A) and Set 2 publications (Groups A and B) combined. *N* indicates the number of publications.

### Structured interview results

Given the unexpected results showing no evidence that the algorithm was helpful, we recruited three participants from Group A and three from Group B for structured interviews as outlined in Supplemental File S3. We selected these participants because their determinations were more often in the minority when compared to other participants. The goals of the interviews were to identify sources of confusion and ambiguity and to identify changes that could improve the algorithm. Topics of the interviews included the expectations and experience of participants before the study and their experiences during the process of making TS and TR determinations. In each interview, we also reviewed a selection of publications for which the participant’s determinations were in the minority.

One source of confusion was an overemphasis in the algorithm that TR relates to a single specific indication or disease, whereas TS relates more broadly to processes or application in multiple indications or diseases. In evaluating TR, it was not clear whether a study had to demonstrate a successful transition from one translational stage to the next or whether progress within a translational stage was sufficient.

None of the six interviewed participants had a background in laboratory science, and all agreed that the lack of such expertise made it difficult to determine TR and TS in laboratory-based publications. In determining TS, it was challenging to assess the novelty of a new method, approach, platform, or tool unless the publication emphasized this point. The extent to which TS must demonstrate that the novel method, approach, platform, or tool could be disseminated and has the desired effect on the conduct or outcomes of TR was unclear. Another source of ambiguity was whether TS requires a formal process of observations, hypothesis, experimentation, data analysis, and conclusions. While these elements were at least implicit in publications reporting original research, they were not present in other publication types. Lastly, a TR publication involving a new method, approach, platform, or tool could stand as a use case for other targets or diseases, but readers could easily miss the TS implications unless the authors emphasize them.

## Discussion

The results of this study showed variable agreement rates and kappa coefficients in the determination of TS and TR in the publications selected for our study. The kappa coefficients in one of the three sets of TS determinations showed substantial agreement, while the other two showed fair and only slight agreement. Two of the three sets of TR determinations showed fair agreement, and one showed only slight agreement. The overall median kappa coefficients for the determinations of TS and TR were 0.39 and 0.22, respectively, both showing only fair agreement (Figure 1). We found no evidence that the algorithm helped to improve agreement rates.

The concept of TS has evolved over time. When the CTSA program was first announced, Elias Zerhouni described it as “an emerging discipline that encompasses both the acquisition of new knowledge about health and disease prevention, preemption, and treatment and the methodologic research necessary to develop or improve research tools” [36]. This definition was used in the earliest CTSA NOFOs [37,38]. Later, NCATS described “research in the science of translation, to discover the mechanistic and operational principles of the intervention development and dissemination process, thereby providing the scientific foundation for improvements in translational efficiency that will accelerate the realization of interventions that improve human health” [39].

In 2021, NCATS formalized definitions of TR and TS, with TS delineated as a “field of investigation focused on understanding the scientific and operational principles underlying each step of the translational process” [1]. After publication of the NCATS 2022 guiding principles [4], discussions during CTSA Program and Enterprise Committee Meetings and a commentary identified continued challenges in defining TS when considering Element E and pilot project applications [40–43]. One notable effort to clarify the distinction between TS and TR was initiated by the CTSA Reviewer Exchange Consortium (CEREC), a collaboration between 9 hubs, including ITHS, that facilitates cross-institution peer reviewer matching for pilot applications. In 2023, CEREC set out to understand the distinction between TS and TR. Pilot Program administrators from 12 CTSA hubs generated a list of questions related to TS principles and used the responses to these questions to evaluate 26 CTSA-funded pilot studies. The objective was to define characteristics that unambiguously differentiated TS and TR as mutually exclusive categories. From an initial list of 24 yes/no questions, the study team identified seven items associated with two principles – “generalizable solutions” and “efficiency and speed” – that appeared to set TS projects apart from TR projects. The authors concluded that CTSA programs might consider these features necessary, though not sufficient, in selecting projects to fund.

The CEREC study had limitations, chief among them the use of proposals for TR pilot projects (i.e., TS was not an explicit goal) and lack of a “gold standard” for defining TS. An expert panel adjudicated the projects as *either* TS or TR (in contrast to the current study) by consensus, permitting only a single dissenter. Although the panel evaluated 26 project proposals in the factor analysis, only 12 (6 TS, 6 TR) were used to examine the factor utility in distinguishing the translational categories. The expert panel could not reach a consensus on the categorization of the remaining 14 proposals, noting that many could have been considered TS if they had been presented with more explicit framing.

The ambiguity of “generalizable solutions” and the difficulty of measuring “efficiency and speed” limits the utility of these concepts to distinguish between TS and TR, though specific questions within each factor offer clearer criteria. Yet generalizability and efficiency must also be viewed in the context of tradeoffs across the research lifecycle, including rigor, reproducibility, participant safety, acceptability, and usability. TS demands systems thinking [45] including careful consideration of how accelerating one step of the research process could produce proximal or downstream effects that limit generalizability, access, and uptake of effective health interventions. Indeed, the CEREC publication noted that all NCATS principles “may be of value for evaluating the fundability of proposed research projects” [44].

In 2024, NCATS updated its interpretation to emphasize intervention and outcomes, stating that TS (emphasis added) “addresses longstanding scientific and operational challenges along the translational science spectrum *through innovations that transform the way research is conducted*, making it faster, more efficient, and more impactful” [2]. As the field matures, we expect the boundaries of TS and TR may continue to evolve. In time, permutations may yield a clearer shared understanding across the CTSA program and health sciences broadly, though the changes may initially provoke further ambiguity as investigators grapple with new interpretations.

Our study has notable limitations. First, we recruited a convenience sample of volunteers, and we relied on agreement rates as an indicator of success in our study because the concept of TS has evolved over time and because the field has not yet developed mature gold standards. The small number of participants limits the precision of the kappa coefficients. The use of volunteers yielded a skew towards participants with experience in clinical research (especially clinical trials) and clinical implementation, and only one participant reported experience in translation from basic to clinical research. Translational stage and research discipline were self-reported, and we did not consider participants’ publication records, funding, peer review experience, or other specific qualifications. We did not ask participants about their motivation to volunteer for the study. All participants had some level of current or prior engagement with ITHS, and most were active contributors to our UL1 research activities, thus having at least some familiarity with the concepts of TR and TS.

Second, we initially limited our assessment to publications arising from our UL1 award. Some participants were co-authors on selected publications, potentially biasing their evaluation of TR and TS. Given our motivation to help investigators identify TS in a project and our focus on UL1-supported publications, very few we selected for this study were limited to TR only. NCATS revised its definition of TS [1,2] after our initial selection of publications but before the participants’ assessments. Publications selected for inclusion in the study were not re-evaluated by the initial screeners (PJM and MV). Since the instructions and algorithm were updated before distribution to participants, this change should not have affected their evaluations. The effort to distinguish between TS and TR in publications was likely more difficult than it would have been in reviewing project proposals, which typically give greater emphasis to innovation, efficiency, and research processes, whereas publications emphasize the experimental results, impact, and future directions. Proposals responding to a request for applications for TS studies would have been more explicit about TS and would likely have been easier for study participants to classify.

The unexpected results of our study prompted consideration of what we could have done to facilitate better agreement rates. Several participants noted that the mental gymnastics of evaluating each publication for TS and TR simultaneously made the task more difficult than it might have been if they had been asked to evaluate only TS. More rigorous, iterative preliminary development could have improved the algorithm. For example, we could have clarified TS requires original research and that TR does not require successful transition from one step to the next in the translational process. From interviews, we learned that the logical process presented concisely on a single page of text in the algorithm was not as intuitive as we had thought. Agreement rates might have been better if the algorithm included more expansive text to highlight the key differences between TS and TR and if we had asked participants to complete the algorithm checklist.

From these insights, we considered whether to repeat the study and evaluate potential improvements from lessons learned. We decided not to do so primarily because the distinction between TS and TR may not be particularly important in evaluating publications describing work that has already been completed. Authors, journal reviewers, and editors readily recognize TR, but it is not clear that they recognize TS or give much consideration to the distinction between TS and TR, and the presence or absence of TS is not a salient concern for most readers. Indeed, conflation of TR and TS is prevalent across scholarly journals and peer-reviewed publications. For instance, published “TS” scores and bibliometric measures have focused on evaluating TR projects within and across fields, without consideration for the application of methods and processes to address barriers to research conduct broadly [46,47]. However, the distinction is vital for investigators responding to the NCATS mission and for reviewers evaluating TS project proposals. Reviewers may especially struggle to distinguish TR and TS in areas that are not familiar to them. The low agreement rates in the assessment of laboratory-based publications in our study emphasizes the importance of subject matter expertise in the recognition of TS.

The words “translational” and “science” have everyday meanings, but “translational science” is a term of art that has a precise, specialized meaning within a specific field. While the individual terms could suggest what the compound term “translational science” means, such intuitive interpretations will inevitably miss the mark, and stand-alone, shortcut explanations such as “generalizable solutions” are too terse, ambiguous, and subjective to be helpful.

**Figure 2.**
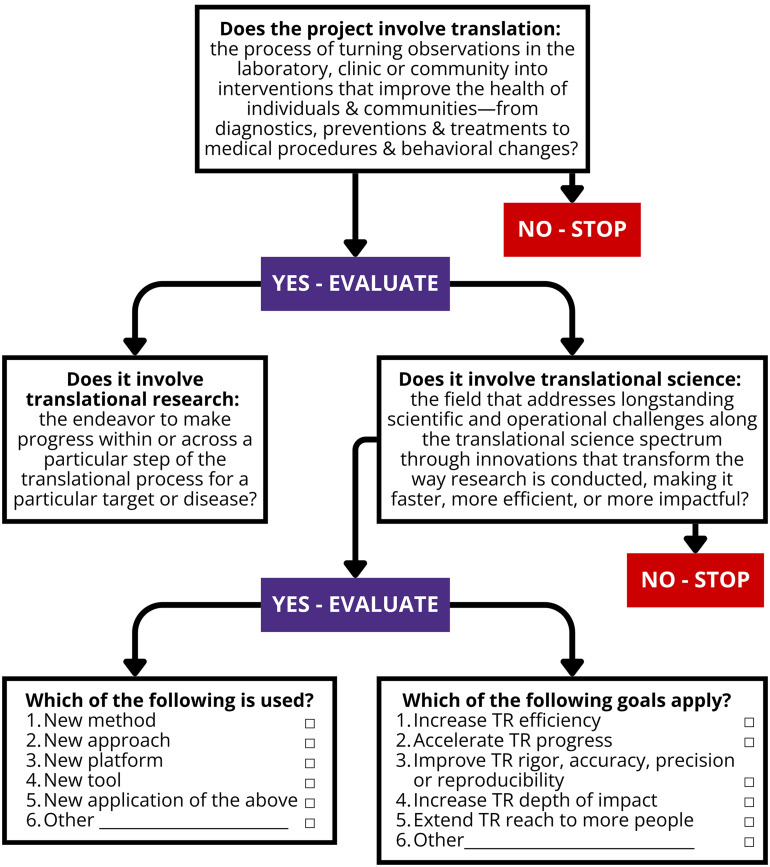
Figure 2 long description. Proposed flow diagram to support identification of TS in research studies. Investigators and reviewers can use the flow diagram to evaluate the role of TS in a proposed project. Definitions of “translational,” “translational research,” and “translational science” originated from PAR-24-272 released on September 4, 2024 [2]. Options listed in the two boxes at the bottom originated from the algorithm used in the current study.

We used our experience in this study to propose a revised, yet to be tested, algorithm that presents a simple flow diagram incorporating the definitions of “translational,” “translational research,” and “translational science” currently formulated by NCATS, with checkboxes to identify the new method, approach, platform, or tool used in the study and articulating how the results of a proposed project are intended to facilitate the conduct or outcomes of TR (Figure 2). The extent to which TS should at least attempt to demonstrate that the novel method, approach, platform, or tool could be disseminated and has the desired effect on the conduct or outcomes of TR remains unclear. Supplemental File S6 provides a 2-page tool with explanatory material from the current UM1 NOFO (PAR-24-272 [2]) and a copy of Figure 2 to be considered by investigators proposing CTSA pilot and Element E projects and by reviewers in assessing proposals for TS projects. Since the algorithm is currently untested, careful evaluation of its performance will be required before widespread adoption, considering the potential for different outcomes based on the users’ prior experience in TS. The algorithm will likely require periodic updates as NCATS refines its terminology. Testing the algorithm across multiple CTSA hubs would provide valuable insights towards understanding TS perspectives in the consortium.

## Supporting information

10.1017/cts.2026.10753.sm001 Martin et al. supplementary material Martin et al. supplementary material

## Supplementary material

The supplementary material for this article can be found at https://doi.org/10.1017/cts.2026.10753.

## Table 1. Long description

The table presents data on 28 study participants, with columns for all participants, Group A only, and Group B only. Data rows include categories for role, translational stage, research discipline, and years of experience. The role distinguishes between staff and faculty, with 5 staff and 14 faculty participants. The translational stage lists categories Preclinical, Clinical Research, Clinical Implementation, Public Health, and Other, with varying numbers of participants in each stage with the highest counts in Clinical Research and/or Clinical Implementation. The research discipline covers areas like Method or process development, Translation from basic to clinical research, Biomedical or health informatics, Health outcomes, services, effectiveness, Clinical epidemiology, Clinical trials, Digital health, mobile health, social media, Pediatrics, Behavioral or social science, and Other; Clinical trials has the highest count. The years of experience shows the mean years of experience and standard deviation for all participants (21 ± 11), Group A (22 ± 14), and Group B (19 ± 7).

Navigate back to Table 1..

## Figure 1. Long description

A horizontal dot plot compares kappa coefficients for translational science and translational research assessments across 42 assessments. The x-axis represents ordered study numbers, while the y-axis shows kappa coefficient values ranging from less than expected to almost perfect. Each blue dot represents a study’s kappa coefficient, with clusters indicating varying levels of agreement. The red dashed line marks the median kappa coefficient for each category. The plot for translational science shows higher coefficients, with many studies reaching almost perfect agreement, while translational research shows a wider spread with lower median values. All values are approximated.

Navigate back to Figure 1..

## Table 2. Long description

The table presents data on the agreement and confidence scores for the determination of TS and TR across different groups and sets. It includes median and overall percentages of agreement, kappa scores, and confidence scores for Group A set 1, Group A set 2, and Group B set 2. Notable trends include lower agreement in TS determinations for Group A set 2 compared to Group A set 1, with median kappa scores of 0.22 and 0.60 respectively. Group B set 2 did not show statistically significant differences in agreement compared to Group A set 2. Overall kappa coefficients for TS determinations were 0.61 for Group A set 1, 0.33 for Group A set 2, and 0.18 for Group B set 2. The table also includes data on TR agreement and confidence scores, with median and overall percentages, kappa scores, and confidence scores provided for each group and set.

Navigate back to Table 2..

## Table 3. Long description

The table presents data on agreement and kappa coefficients for various publication subject matters in translational science and research. It includes six subject matters: Biostatistics, EHR, RCS, Ethics, Laboratory, and Other. Each subject matter lists the number of publications (N), agreement range, and kappa range for both translational science and translational research. Biostatistics shows perfect agreement in translational science but lower agreement in translational research. EHR and RCS show moderate agreement in both areas. Ethics shows lower agreement in translational science but higher in translational research. Laboratory shows the lowest agreement in translational science and moderate agreement in translational research. Other subject matters show moderate agreement in both areas. The table highlights variations in agreement and kappa coefficients across different subject matters and research types.

Navigate back to Table 3..

## Figure 2. Long description

Flowchart evaluating translational science in research projects. The process begins by determining if the project involves translation, defined as turning observations into health interventions. If no, the process stops. If yes, it proceeds to evaluate if the project involves translational research, which aims to progress within a specific step of the translational process for a particular target or disease. If no, the process stops. If yes, it further evaluates if the project involves translational science, addressing scientific and operational challenges to make research faster, more efficient, or more impactful. If no, the process stops. If yes, it identifies the methods used, such as new methods, approaches, platforms, tools, or applications. Additionally, it lists goals like increasing efficiency, accelerating progress, improving rigor, increasing depth of impact, extending reach, or addressing other objectives.

Navigate back to Figure 2..
