# Supplementary material for: Steps toward developing an algorithm to facilitate recognition of translational science
Source: J Clin Transl Sci. 2026 May 15;10(1):e100. doi: 10.1017/cts.2026.10753 (PMC13312354; doi:10.1017/cts.2026.10753)

**SUPPLEMENT**

**File S1: INVITATION AND INSTRUCTIONS FOR TS & TR DETERMINATIONS**

**First Round**

**Second Round**

**File S2: THE ORIGINAL ALGORITHM**

**File S3: INTERVIEW GUIDE**

**File S4: STUDY DATA**

**File S5: FACULTY AGREEMENT SCORES FOR DETERMINATION OF TS AND TR**

**File S6: TOOL PROPOSED FOR FUTURE EVALUATION**

**SUPPLEMENTAL FILE S1: INVITATION/INSTRUCTIONS FOR TS & TR DETERMINATIONS**

**Instructions—First Round**

Thank you for volunteering to participate in our ITHS project titled, “Translational Science: Do We Recognize It When We See It?”

Attached are links to 14 publications, many authored by ITHS faculty members. Your task is to review each of these publications to determine whether it represents translational science (yes or no) and whether it represents translational research (yes or no). These are independent determinations, since a publication could represent both, neither, or one but not the other.

For reference, NCATS has defined translational science as follows:

“Translational science is the field that addresses longstanding scientific and operational challenges along the translational science spectrum through innovations that transform the way research is conducted, making it faster, more efficient, and more impactful.”

We have a few suggestions that might be helpful as you review these publications.

- At your discretion, you may consult other resources to explore the meaning of “translational science,” but no such effort on your part is expected or required.
- It is not necessary to read through the entire publication to answer the two questions. Just scan through the text until you reach the point that you have seen enough information to draw your conclusions.
- We ask that you take the task seriously, but do not overthink your answers. This exercise is not a test, and you will not receive a grade. It should be possible to reach your conclusions within no more than 5 to 10 minutes per publication.
- You are free to change your answers as you go through the exercise. Your answers are not final until you submit your ballot.
- We ask that you answer both questions for all 14 publications, even if you are not sure about your answers.
- For each publication, two additional questions will ask about your level of confidence that your answers will agree with the answers submitted by others.

Within the next few days, we will circulate a poll to identify a date and time for a group meeting to review the results of this round and to participate in the development of a tool that will be used to help others recognize translational science when they see it.

**Instructions—Second Round**

Thank you for volunteering to participate in our ITHS project titled, “Translational Science: Do We Recognize It When We See It?”

Attached are links to 14 publications, many authored by ITHS faculty members. Your task is to review each of these publications to determine whether it represents translational science (yes or no) and whether it represents translational research (yes or no). These are independent determinations, since a publication could represent both, neither, or one but not the other.

From our experience in the first round of reviews, we have developed and refined a tool that we believe will be helpful in assessing translational science and translational research in publications. We ask that you review this tool before you begin your reviews of the 14 publications, and we suggest that you follow the algorithm outlined in the tool as you consider your assessments.

We have a few suggestions that might be helpful as you review these publications.

- It is not necessary to read through the entire publication to answer the two questions. Just scan through the text until you reach the point that you have seen enough information to draw your conclusions.
- We ask that you take the task seriously, but do not overthink your answers. This exercise is not a test, and you will not receive a grade. It should be possible to reach your conclusions within no more than 5 to 10 minutes per publication.
- You are free to change your answers as you go through the exercise. Your answers are not final until you submit your ballot.
- We ask that you answer both questions for all 14 publications, even if you are not sure about your answers.
- For each publication, two additional questions will ask about your level of confidence that your answers will agree with the answers submitted by others.

**SUPPLEMENTAL FILE S2: THE ORIGINAL ALGORITHM**

Translational research and translational science are not mutually exclusive. Any given translational health project or publication can involve both or one but not the other. If both are involved, one could be primary and the other secondary**.**

- **Translational research is directed to a single specific indication or disease.**
- **Translational science is directed more broadly to the processes involved in translational research or to their application for multiple indications or diseases**.

The following steps will help recognize translational research and translational science in projects or publications.

1. Is the activity translational, i.e., an activity broadly intended to develop and evaluate interventions (e.g., drugs, devices, life-style changes, institutional policies) that can be applied to improve the health of individual humans or the public?

☐ IF NO, stop here. The activity is not translational, although it may have other worthy purposes (e.g., education).

☐ IF YES, continue below.

1. Does the activity involve translational research, i.e., a process that turns observations in the laboratory, clinic or community into a new specific intervention intended to improve the health of individuals or the public?

☐ IF YES, the activity involves translational research.

☐ IF NO, the activity does not involve translational research, but it may involve translational science.

Continue below.

1. Does the activity involve translational science?
2. Does the activity seek broadly to improve the translational research process through the development of any of the following or anything like one of the following?
3. New methods ☐
4. New approaches ☐
5. New platforms ☐
6. New tools ☐
7. New applications of the above ☐

☐ IF NO, stop here. The activity does not involve translational science.

☐ IF YES, identify those that apply and continue below.

1. Does the activity seek broadly to improve the translational research process by producing any of the following outcomes?
2. Increased efficiency ☐
3. Accelerated progress ☐
4. Improved rigor or reproducibility ☐
5. Increased depth of impact ☐
6. Extended reach to improve health equity ☐

☐ IF NO, stop here. The activity does not involve translational science.

☐ IF YES, identify those that apply and conclude that the activity involves translational science.

**SUPPLEMENTAL FILE S3: INTERVIEW GUIDE**

*This guide was used for one-on-one interviews with participants whose assessments diverged from the majority decision more frequently than other participants.*

**Introductory Information**

1. Thank you for participating in the original study activities and for agreeing to this interview
2. Summary of the results so far: agreement rates much lower than expected and algorithm not helpful
3. Participants selected for interviews were more often in the minority
4. Goals of the interview: identify sources of confusion, improve the algorithm

**Expectations/experience before participation**

1. What were the participant’s main motivations for volunteering for the project?
   1. Desire to contribute to an interesting project
   2. Opportunity to test participant’s understanding of the distinction between TS and TR
   3. Demonstrate participant’s own good understanding
   4. Desire to get a better understanding of the distinction
   5. Other

**Level of understanding**

1. In your own words, how would you describe: (a) translational research, (b) translational science?
2. How did you arrive at this understanding? (e.g., prior exposure to or work in area, discussions with colleagues)?

**Experience during participation**

1. How did the difficulty of making the TS and TR evaluations compare with the participant’s expectations: easier, more difficult, about the same? If more difficult, what made it difficult?
2. In your experience, was the algorithm helpful? If not, why not?

**Minority Determinations made by the participant**

1. Read through summary determinations as applicable. Identify and discuss any points of ambiguity, confusion or disagreement.

**Algorithm Review**

1. Read through the algorithm to solicit suggestions that could clarify the guidance.

**Thank You and Next Steps**

1. Possible next steps
   1. Interest in another round of testing?
   2. Interest in contributing to the manuscript?
   3. Other suggestions for dissemination of learning?

| **SUPPLEMENTAL FILE S4: STUDY DATA**  **Group A Set 1 Translational Science Assessments** | | | | **Participant** | | | | | | | | | |  |  |
| --- | --- | --- | --- | --- | --- | --- | --- | --- | --- | --- | --- | --- | --- | --- | --- |
| **Reference*** | **PMID** | **Type** | **Subject** | **1** | **2** | **3** | **4†** | **5** | **6** | **7†** | **8** | **9** | **10** | **Agreement** | **Kappa** |
| 1 | 23253665 | Review | RCS | Y | Y | Y | Y | Y | Y | Y | Y | Y | Y | 100 | 1.00 |
| 2 | 33945415 | Perspective | Ethics | Y | Y | Y | Y | Y | Y | Y | Y | Y | Y | 100 | 1.00 |
| 3 | 27057344 | OR Plan | Ethics | N | N | N | N | N | N | Y | N | N | N | 80 | 0.60 |
| 4 | 35774016 | OR | Biostat | Y | Y | Y | Y | Y | Y | Y | Y | Y | Y | 100 | 1.00 |
| 5 | 34633425 | OR | EHR | N | Y | Y | Y | Y | Y | Y | Y | Y | Y | 80 | 0.60 |
| 6 | 34268525 | OR | EHR | Y | Y | Y | Y | Y | Y | Y | Y | N | Y | 80 | 0.60 |
| 7 | 32735337 | OR | Ethics | Y | Y | Y | Y | N | Y | Y | Y | Y | Y | 80 | 0.60 |
| 8 | 34367672 | OR | RCS | Y | Y | Y | N | Y | Y | Y | Y | Y | Y | 80 | 0.60 |
| 9 | 38935715 | OR | Lab | N | Y | N | N | N | N | N | Y | N | Y | 53 | 0.07 |
| 10 | 39208112 | OR | Lab | Y | Y | N | N | N | Y | Y | N | Y | Y | 47 | -0.07 |
| 11 | 34586386 | OR | EHR | Y | Y | Y | Y | Y | Y | Y | Y | Y | Y | 100 | 1.00 |
| 12 | 32883156 | OR | Other | Y | Y | Y | Y | Y | Y | Y | Y | Y | Y | 100 | 1.00 |
| 13 | 39172838 | OR | Lab | N | N | N | N | Y | N | N | N | N | N | 80 | 0.60 |
| 14 | 39236156 | OR | Lab | Y | Y | Y | Y | Y | N | Y | N | N | N | 47 | -0.07 |
| Abbreviations: OR, original research; RCS, representative cohort selection; EHR, electronic health records  *Numbering does not match the manuscript.  †Staff member | | | | | | | | | | | | |  |  |  |

| **Group A Set 2 Translational Science Assessments** | | | | **Participant** | | | | | | | | | |  |  |
| --- | --- | --- | --- | --- | --- | --- | --- | --- | --- | --- | --- | --- | --- | --- | --- |
| **Reference*** | **PMID** | **Type** | **Subject** | **1** | **2** | **3** | **4†** | **5** | **6** | **7†** | **8** | **9** | **10** | **Agreement** | **Kappa** |
| 15 | 39298590 | OR | Lab | N | Y | N | Y | Y | N |  | Y | N | N | 44 | -0.11 |
| 16 | 39208111 | OR | Lab | N | Y | Y | Y | Y | N |  | Y | N | Y | 50 | 0.00 |
| 17 | 38963839 | OR | Lab | N | Y | Y | Y | Y | Y |  | N | N | N | 44 | -0.11 |
| 18 | 38935717 | OR | Lab | N | Y | Y | Y | Y | N |  | N | N | Y | 44 | -0.11 |
| 19 | 36751464 | OR | EHR | Y | Y | Y | Y | Y | Y |  | Y | Y | Y | 50 | 1.00 |
| 20 | 35227445 | OR | EHR | Y | Y | Y | N | Y | Y |  | Y | Y | Y | 78 | 0.56 |
| 21 | 34908169 | Position Paper | RCS | Y | N | Y | Y | Y | Y |  | Y | Y | Y | 78 | 0.56 |
| 22 | 34192059 | Position Paper | Ethics | Y | Y | Y | Y | N | Y |  | Y | N | Y | 61 | 0.22 |
| 23 | 34074706 | Position Paper | Ethics | Y | N | N | Y | Y | Y |  | Y | N | N | 44 | -0.11 |
| 24 | 33825622 | Commentary | Ethics | Y | N | Y | Y | Y | Y |  | Y | N | Y | 61 | 0.22 |
| 25 | 33290878 | OR | RCS | Y | Y | Y | Y | Y | Y |  | Y | Y | N | 78 | 0.56 |
| 26 | 32638010 | OR | EHR | Y | Y | Y | Y | Y | Y |  | Y | Y | Y | 100 | 1.00 |
| 27 | 32128451 | OR | Other | Y | N | Y | Y | Y | Y |  | Y | N | N | 50 | 0.00 |
| 28 | 31823406 | OR | Biostat | Y | Y | Y | Y | Y | Y |  | Y | Y | Y | 100 | 1.00 |
| Abbreviations: OR, original research; RCS, representative cohort selection; EHR, electronic health records  *Numbering does not match the manuscript.  †Staff member | | | | | | | | | | | | |  |  |  |

| **Group B Set 2 Translational Science Assessments** | | | | **Participant** | | | | | | | | |  |  |  |
| --- | --- | --- | --- | --- | --- | --- | --- | --- | --- | --- | --- | --- | --- | --- | --- |
| **Reference*** | **PMID** | **Type** | **Subject** | **11†** | **12** | **13** | **14** | **15** | **16†** | **17†** | **18** | **19** |  | **Agreement** | **Kappa** |
| 15 | 39298590 | OR | Lab | N | N | N | N | Y | N | Y | Y | Y |  | 44 | -0.11 |
| 16 | 39208111 | OR | Lab | Y | N | N | N | Y | N | Y | Y | N |  | 44 | -0.11 |
| 17 | 38963839 | OR | Lab | Y | N | N | Y | N | N | Y | Y | Y |  | 44 | -0.11 |
| 18 | 38935717 | OR | Lab | Y | N | N | Y | N | N | Y | Y | N |  | 44 | -0.11 |
| 19 | 36751464 | OR | EHR | Y | Y | Y | Y | Y | Y | Y | Y | Y |  | 100 | 1.00 |
| 20 | 35227445 | OR | EHR | Y | N | Y | Y | Y | N | Y | Y | Y |  | 61 | 0.22 |
| 21 | 34908169 | Position Paper | RCS | Y | Y | Y | Y | Y | Y | Y | Y | N |  | 78 | 0.56 |
| 22 | 34192059 | Position Paper | Ethics | Y | N | N | Y | Y | Y | Y | Y | N |  | 50 | 0.00 |
| 23 | 34074706 | Position Paper | Ethics | Y | Y | N | Y | Y | Y | N | N | N |  | 44 | -0.11 |
| 24 | 33825622 | Commentary | Ethics | Y | Y | N | N | Y | Y | N | N | N |  | 44 | -0.11 |
| 25 | 33290878 | OR | RCS | Y | N | Y | Y | Y | N | N | Y | N |  | 44 | -0.11 |
| 26 | 32638010 | OR | EHR | Y | N | Y | Y | Y | N | Y | Y | N |  | 50 | 0.00 |
| 27 | 32128451 | OR | Other | Y | Y | Y | N | Y | Y | Y | Y | Y |  | 78 | 0.56 |
| 28 | 31823406 | OR | Biostat | Y | Y | Y | Y | Y | Y | Y | Y | Y |  | 100 | 1.00 |
| Abbreviations: OR, original research; RCS, representative cohort selection; EHR, electronic health records  *Numbering does not match the manuscript. | | | | | | | | | | | | |  |  |  |

†Staff member

| **Group A Set 1 Translational Research Assessments** | | | | **Participant** | | | | | | | | | |  |  |
| --- | --- | --- | --- | --- | --- | --- | --- | --- | --- | --- | --- | --- | --- | --- | --- |
| **Reference*** | **PMID** | **Type** | **Subject** | **1** | **2** | **3** | **4†** | **5** | **6** | **7†** | **8** | **9** | **10** | **Agreement** | **Kappa** |
| 1 | 23253665 | Review | RCS | N | N | N | Y | N | N | N | N | N | N | 80 | 0.60 |
| 2 | 33945415 | Perspective | Ethics | N | N | N | Y | N | N | N | N | N | N | 80 | 0.60 |
| 3 | 27057344 | OR Plan | Ethics | Y | Y | Y | N | Y | Y | Y | Y | Y | Y | 80 | 0.60 |
| 4 | 35774016 | OR | Biostat | N | Y | N | N | N | N | Y | N | N | N | 61 | 0.22 |
| 5 | 34633425 | OR | EHR | Y | Y | Y | N | Y | N | Y | Y | Y | N | 53 | 0.07 |
| 6 | 34268525 | OR | EHR | N | Y | N | N | Y | Y | Y | N | Y | N | 44 | -0.11 |
| 7 | 32735337 | OR | Ethics | N | Y | N | Y | Y | Y | Y | N | N | N | 44 | -0.11 |
| 8 | 34367672 | OR | RCS | N | Y | N | Y | N | N | Y | N | N | N | 53 | 0.07 |
| 9 | 38935715 | OR | Lab | Y | Y | Y | N | Y | Y | Y | y | Y | Y | 80 | 0.60 |
| 10 | 39208112 | OR | Lab | N | Y | Y | N | Y | Y | Y | Y | N | N | 47 | -0.07 |
| 11 | 34586386 | OR | EHR | N | Y | N | N | N | N | Y | N | N | N | 64 | 0.29 |
| 12 | 32883156 | OR | Other | N | Y | Y | Y | Y | Y | Y | N | N | N | 47 | -0.07 |
| 13 | 39172838 | OR | Lab | Y | Y | Y | Y | N | Y | Y | Y | Y | Y | 80 | 0.60 |
| 14 | 39236156 | OR | Lab | N | Y | Y | Y | N | Y | Y | Y | Y | Y | 64 | 0.29 |
| Abbreviations: OR, original research; RCS, representative cohort selection; EHR, electronic health records  *Numbering does not match the manuscript. | | | | | | | | | | | |  |  |  |  |

†Staff member

| **Group A Set 2 Translational Research Assessments** | | | | **Participant** | | | | | | | | | |  |  |
| --- | --- | --- | --- | --- | --- | --- | --- | --- | --- | --- | --- | --- | --- | --- | --- |
| **Reference*** | **PMID** | **Type** | **Subject** | **1** | **2** | **3** | **4†** | **5** | **6** | **7†** | **8** | **9** | **10** | **Agreement** | **Kappa** |
| 15 | 39298590 | OR | Lab | Y | Y | Y | Y | N | Y |  | N | Y | Y | 61 | 0.22 |
| 16 | 39208111 | OR | Lab | Y | Y | Y | Y | N | Y |  | N | Y | Y | 61 | 0.22 |
| 17 | 38963839 | OR | Lab | Y | Y | Y | Y | Y | Y |  | Y | Y | Y | 100 | 1.00 |
| 18 | 38935717 | OR | Lab | Y | N | Y | Y | N | Y |  | Y | Y | Y | 61 | 0.22 |
| 19 | 36751464 | OR | EHR | Y | Y | N | N | N | N |  | N | N | N | 61 | 0.22 |
| 20 | 35227445 | OR | EHR | Y | Y | N | Y | N | N |  | N | Y | Y | 44 | -0.11 |
| 21 | 34908169 | Position Paper | RCS | Y | Y | N | N | N | N |  | N | N | Y | 50 | 0.00 |
| 22 | 34192059 | Position Paper | Ethics | Y | Y | N | N | Y | N |  | N | N | N | 50 | 0.00 |
| 23 | 34074706 | Position Paper | Ethics | Y | Y | N | N | N | N |  | N | N | N | 61 | 0.22 |
| 24 | 33825622 | Commentary | Ethics | Y | Y | N | N | Y | N |  | N | N | Y | 44 | -0.11 |
| 25 | 33290878 | OR | RCS | Y | Y | Y | N | Y | N |  | N | N | N | 44 | -0.11 |
| 26 | 32638010 | OR | EHR | Y | Y | N | Y | N | N |  | N | N | N | 50 | 0.00 |
| 27 | 32128451 | OR | Other | Y | Y | Y | Y | Y | N |  | N | N | N | 44 | -0.11 |
| 28 | 31823406 | OR | Biostat | Y | Y | N | Y | Y | N |  | N | N | N | 44 | -0.11 |
| Abbreviations: OR, original research; RCS, representative cohort selection; EHR, electronic health records  *Numbering does not match the manuscript.  †Staff member | | | | | | | | | | | |  |  |  |  |

| **Group B Set 2 Translational Research Assessments** | | | | **Participant** | | | | | | | | |  |  |  |
| --- | --- | --- | --- | --- | --- | --- | --- | --- | --- | --- | --- | --- | --- | --- | --- |
| **Reference*** | **PMID** | **Type** | **Subject** | **11†** | **12** | **13** | **14** | **15** | **16†** | **17†** | **18** | **19** |  | **Agreement** | **Kappa** |
| 15 | 39298590 | OR | Lab | Y | N | Y | Y | Y | Y | Y | Y | Y |  | 78 | 0.56 |
| 16 | 39208111 | OR | Lab | Y | N | Y | Y | Y | Y | Y | Y | Y |  | 78 | 0.56 |
| 17 | 38963839 | OR | Lab | Y | N | Y | N | Y | Y | Y | Y | Y |  | 61 | 0.22 |
| 18 | 38935717 | OR | Lab | Y | Y | Y | N | Y | Y | N | Y | N |  | 50 | 0.00 |
| 19 | 36751464 | OR | EHR | Y | Y | N | N | N | N | N | N | N |  | 61 | 0.22 |
| 20 | 35227445 | OR | EHR | N | Y | N | N | N | Y | N | Y | Y |  | 44 | -0.11 |
| 21 | 34908169 | Position Paper | RCS | N | Y | N | N | N | N | N | N | N |  | 78 | 0.56 |
| 22 | 34192059 | Position Paper | Ethics | N | N | N | N | N | N | N | Y | N |  | 78 | 0.56 |
| 23 | 34074706 | Position Paper | Ethics | N | Y | N | N | N | N | N | N | N |  | 78 | 0.56 |
| 24 | 33825622 | Commentary | Ethics | N | Y | N | N | N | N | N | N | N |  | 78 | 0.56 |
| 25 | 33290878 | OR | RCS | N | N | N | N | N | Y | N | N | N |  | 78 | 0.56 |
| 26 | 32638010 | OR | EHR | N | N | N | N | N | Y | N | N | N |  | 78 | 0.56 |
| 27 | 32128451 | OR | Other | N | Y | N | N | N | N | N | N | Y |  | 61 | 0.22 |
| 28 | 31823406 | OR | Biostat | N | Y | N | N | N | N | N | N | N |  | 78 | 0.56 |
| Abbreviations: OR, original research; RCS, representative cohort selection; EHR, electronic health records  *Numbering does not match the manuscript.  †Staff member | | | | | | | | | | | |  |  |  |  |

| **Supplemental File S5. Faculty Agreement Scores for Determination of TS and TR** | | | | | |
| --- | --- | --- | --- | --- | --- |
| **Outcome** | **Group A Set 1** | **Group A Set 2** | **P-value*** | **Group B Set 2** | **P-Value†** |
| Percent TS agreement, median | 87.5 | 57 | 0.13 | 47 | 0.37 |
| TS kappa, median | 0.75 | 0.14 |  | -0.07 |  |
| TS agreement, overall | 81 | 67 |  | 57 |  |
| TS kappa, overall | 0.62 | 0.33 |  | 0.13 |  |
| Percent TR Agreement, median | 75 | 52 | 0.046 | 67 | 0.012 |
| TR kappa, median | 0.50 | 0.04 |  | 0.33 |  |
| TR agreement, overall | 71 | 54 |  | 65 |  |
| TR kappa, overall | 0.42 | 0.08 |  | 0.30 |  |
| *Group A Set 1 vs. Group A Set 2 | | | | | |
| †Group A Set 2 vs. Group B Set 2 | | | | | |

**SUPPLEMENTAL FILE S6: TOOL PROPOSED FOR FUTURE EVALUATION**

'Translation' is defined by NCATS as the process of turning observations in the laboratory, clinic and community into interventions that improve the health of individuals and communities – from diagnostics, preventions, and treatments to medical procedures and behavioral changes. ‘Translational research’ (TR) is defined by NCATS as the endeavor to traverse a particular step of the translational process for a particular target or disease. ‘Translational science’ (TS) is the field that addresses longstanding scientific and operational challenges along the translational science spectrum through innovations that transform the way research is conducted, making it faster, more efficient, and more impactful. Whereas translational research focuses on the specific case of a target or disease, translational science is focused on the general case that applies to any target or disease; advances in translational science are the focus of this NOFO. A key tenet of translational science is to understand common causes of inefficiency and failure in translational research projects (e.g., incorrect predictions of the toxicity or efficacy of new drugs, lack of data interoperability, ineffective clinical trial recruitment). Many of these causes are the same across targets, diseases, and therapeutic areas; therefore, advances in translational science will increase the efficiency and effectiveness of translational research to enhance health, lengthen life, and reduce the burdens of illness and disability. Like any other science, translational science seeks to elucidate general operative principles to transform translation from an empirical, phenomenological process into a predictive science. The application of scientific and operational innovation and strategies to improve the efficiency and effectiveness of all research is at the heart of developing, demonstrating, and disseminating the science of translation.

An example - for illustration only - to help clarify the relationship between TR and TS follows. An investigator initiates a study evaluating whether a particular drug can improve specific outcomes in diabetes. The investigator could utilize commonly used TR methodology, such as testing effectiveness of the drug using a limited number of traditional clinical endpoints and employing established recruitment methods. By contrast, an investigator could approach from a TS framework. In the diabetes example, the same drug could be evaluated, yet an underlying common problem in TR such as recruitment and retention in underserved communities consistent with the NIH Inclusion Policies, could be addressed at the same time. From this TS perspective, the investigator could include in the study an understanding of the fundamental barriers to recruitment and test an intervention directed at hypothesized causes and mechanisms of the barriers in a particular underserved population. Engagement with this population before protocol development may lead to some interesting potential Patient Reported Outcomes (PRO) that may not only impact recruitment and retention but may also predict future adherence (that could be the focus of a future study). Ultimately, the questions to be answered in this TS version would be if the innovation accomplishes full recruitment and retention of a desired population more effectively and efficiently than without the innovation. Of note in this TS version of the study, the intervention utilized to overcome the recruitment challenges, if determined to be effective, would likely be more generally applicable for other interventions that target not only diabetes but potentially clinical trials in other disease areas where similar recruitment barriers occur. Of further note, although the study itself was focused on a particular disease and drug, its TS version delivers knowledge that is more generally applicable.

Translational research and translational science are not mutually exclusive. A translational health project can involve both or one but not the other.


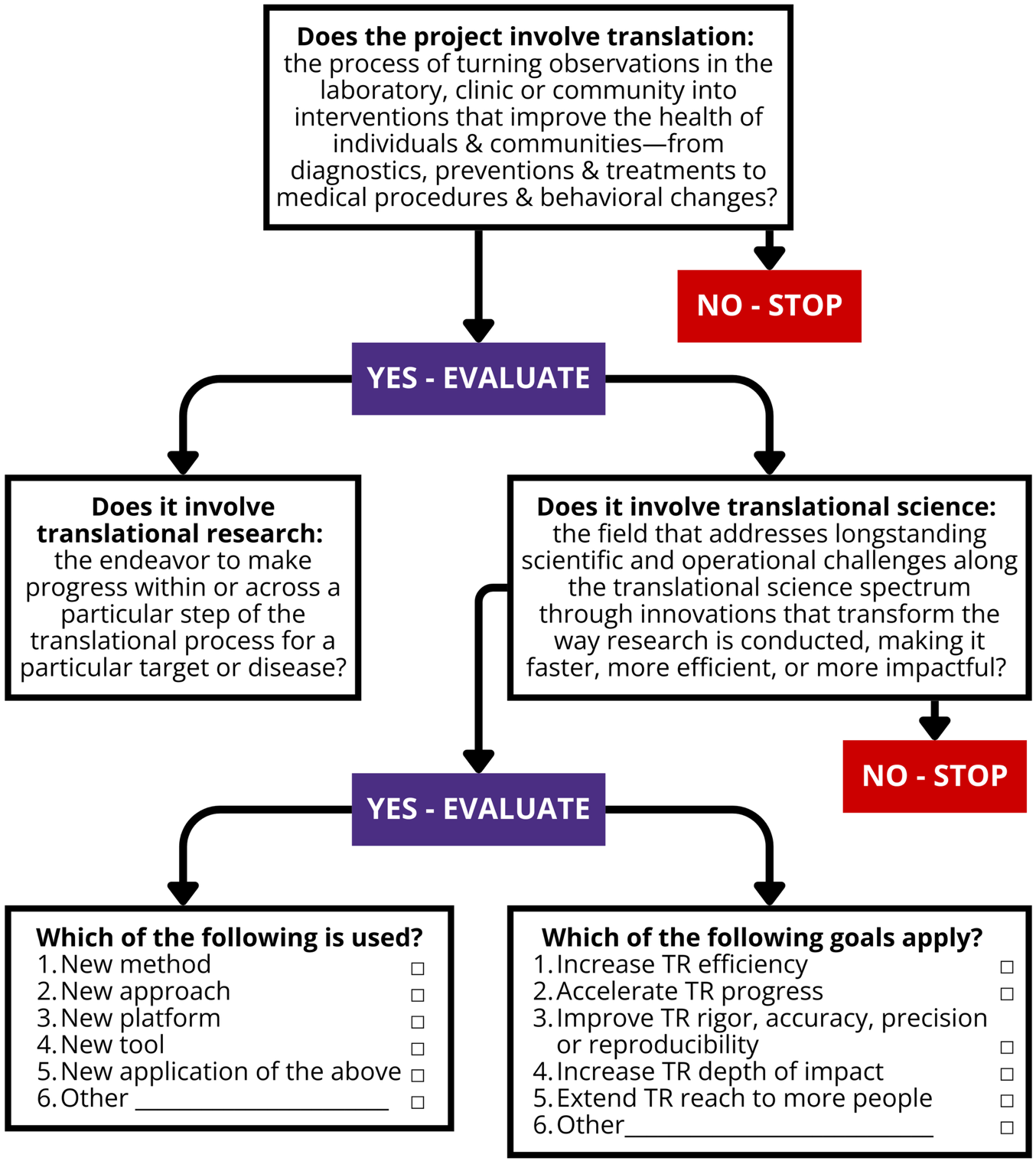

Supplement: Martin et al. supplementary material [file S2059866126107535sup001.docx]
